# A collagen hydrolysate/milk protein-blend stimulates muscle anabolism equivalently to an isoenergetic milk protein-blend containing a greater quantity of essential amino acids in older men

**DOI:** 10.1016/j.clnu.2021.01.002

**Published:** 2021-06

**Authors:** M.S. Brook, P. Scaife, J.J. Bass, J. Cegielski, S. Watanabe, D.J. Wilkinson, K. Smith, B.E. Phillips, P.J. Atherton

**Affiliations:** a MRC-Versus Arthritis Centre for Musculoskeletal Ageing Research and Nottingham NIHR BRC, Clinical, Metabolic and Molecular Physiology, School of Medicine, University of Nottingham, Derby, UK; b School of Life Sciences, University of Nottingham, Derby, UK

**Keywords:** Muscle protein synthesis, Collagen, Exercise, Ageing, Oral nutritional supplement, Essential amino acids, MPS, muscle protein synthesis, MPB, muscle protein breakdown, EAA, essential amino acids, NEAA, non-essential amino acids, BCAA, branched chain amino acids, MP, milk protein, CP, collagen protein hydrolysate, BMI, body mass index, 1-RM, 1 repetition maximum, PVDF, polyvinylidene difluoride, TBST, tris buffered saline/Tween 20, DIAAS, digestible indispensable amino acid score, VL, vastus lateralis, ONS, oral nutritional supplement, FED, feed, FED-EX, feed + exercise

## Abstract

**Background & aims:**

Nutritional composition is key for skeletal muscle maintenance into older age. Yet the acute effects of collagen protein blended with other protein sources, in relation to skeletal muscle anabolism, are ill-defined. We investigated human muscle protein synthesis (MPS) responses to a 20 g blend of collagen protein hydrolysate + milk protein (CP+MP, 125 ml) oral nutritional supplement (ONS) vs. 20 g non-blended milk protein source (MP, 200 ml) ONS, in older adults.

**Methods:**

Healthy older men (N = 8, 71±1 y, BMI: 27±1 kg·m^−2^) underwent a randomized trial of 20 g protein, from either a CP+MP blend (Fresubin®3.2 kcal DRINK), or a kcal-matched (higher in essential amino acids (EAA) ONS of MP alone. *Vastus lateralis* (VL) MPS and plasma AA were determined using stable isotope-tracer mass spectrometry; anabolic signaling was quantified via immuno-blotting in VL biopsies taken at baseline and 2/4 h after ONS feeding. Plasma insulin was measured via enzyme-linked immunosorbent assay (ELISA). Measures were taken at rest, after the feed (FED) and after the feed + exercise (FED-EX) conditions (unilateral leg exercise, 6 × 8, 75% 1-RM).

**Results:**

MP resulted in a greater increase in plasma leucine (MP mean: 152 ± 6 μM, CP+MP mean: 113 ± 4 μM (Feed P < 0.001) and EAA (MP mean: 917 ± 25 μM, CP+MP mean: 786 ± 15 μM (Feed P < 0.01) than CP+MP. CP + MP increased plasma glycine (peak 385 ± 57 μM (P < 0.05)), proline (peak 323 ± 29 μM (P < 0.01)) and non-essential amino acids (NEAA) (peak 1621 ± 107 μM (P < 0.01)) with MP showing no increase. Plasma insulin increased in both trials (CP+MP: 58 ± 10 mU/mL (P < 0.01), MP: 42 ± 6 mU/mL (P < 0.01), with peak insulin greater with CP+MP vs. MP (P < 0.01). MPS demonstrated equivalent increases in response to CP+MP and MP under both FED (MP: 0.039 ± 0.005%/h to 0.081 ± 0.014%/h (P < 0.05), CP+MP: 0.042 ± 0.004%/h to 0.085 ± 0.007%/h (P < 0.05)) and FED-EX (MP: 0.039 ± 0.005%/h to 0.093 ± 0.013%/h (P < 0.01), CP+MP: 0.042 ± 0.004%/h to 0.105 ± 0.015%/h, (P < 0.01)) conditions. FED muscle p-mTOR fold-change from baseline increased to a greater extent with CP+MP vs. MP (P < 0.05), whilst FED-EX muscle p-eEF2 fold-change from baseline decreased to a greater extent with CP+MP vs. MP (P < 0.05); otherwise anabolic signaling responses were indistinguishable.

**Conclusion:**

Fresubin®3.2 kcal DRINK, which contains a 20 g mixed blend of CP+MP, resulted in equivalent MPS responses to MP alone. Fresubin® 3.2 Kcal DRINK may provide a suitable alternative to MP for use in older adults and a convenient way to supplement calories and protein to improve patient adherence and mitigate muscle mass loss.

## 1 Introduction

Maintenance of skeletal muscle mass across the life-course is dependent upon intake of adequate dietary protein; this is because amino acids (AA) within dietary proteins acutely stimulate muscle protein synthesis (MPS) and thereby replenish muscle protein stores lost to muscle protein breakdown (MPB) in times of fasting [1]. As such, intake of the appropriate quantity and quality of dietary proteins is a pre-requisite for skeletal muscle health. The essential AA (EAA) are crucial for the stimulation of MPS in humans, with flooding doses of individual EAA [2] stimulating MPS even in the absence of complete AA mixtures [3]. The anabolic effects of EAA are thought to be due to their signaling properties, for instance, leucine [4] can stimulate MPS in the absence of other EAA (via depletion of endogenous EAA pools) in target tissues such as muscle.

Ageing is associated with gradual declines in skeletal muscle mass due to the loss of muscle fibers as a function of neurodegeneration, and a loss of muscle fiber cross sectional area [5]. A major feature of aged muscle would seem to be impaired handling of dietary proteins; reflecting this, many research groups have shown that older individuals' anabolic response to protein intake is blunted with advancing age [[6], [7], [8]]. Further, despite dietary protein consumption alongside resistance-type exercise being arguably the most effective countermeasure to mitigate age related declines in muscle mass and strength, anabolic deficits with age remain [9]. This, alongside longitudinal trials showing improved muscle maintenance with higher protein intakes [10] is likely one of the major reasons the recommendations for protein intake has recently been increased to 1–1.2 g/kg/day for healthy older adults and 1.2–1.5 g/kg/day for acute or chronic illness [11]. However, protein intake in grams per kilogram, could be quite different depending upon individuals' preferences e.g. for plant, meat or milk derived proteins. The composition of AA in distinct protein sources can differ markedly, as can the ‘biological value’ based upon digestibility, bioavailability and/or tissue utilization [12,13]. Thus, another major area of research in terms of ageing skeletal muscle and dietary protein intake, relates to distinct protein sources and their biological effects [14], in the context of acute muscle protein turnover and muscle mass outcomes - with or without exercise.

There are a number of aspects of this biological value approach that do not get to the crux of being able to generalize protein sources as superior or inferior. For example, leucine content differs among protein sources and has been shown to direct the anabolic response to a greater extent than total protein load [15,16] i.e. leucine content alone could explain the apparent higher biological value. Also, there are absorption aspects with, for example, casein-derived milk proteins apparently clotting and slowing absorption [17]. However, we have shown that rates of aminoacidemia do not impact anabolic responses in muscle [18] and instead, any differences in casein vs. other protein sources more likely reflects the primary AA composition (i.e. leucine content). As such, there are many factors that need taking into consideration with protein provision, particularly in the design of oral nutritional supplements (ONS) that are key in overcoming many forms of malnutrition. The effectiveness of any ONS relies on increasing compliance, with Low volume, high energy dense ONS shown to have greater success [19]. Protein type, such as collagen, may therefore hold strengths in improving the production and palatability of low volume, high energy, high protein ONS [20]. However, protein quality is key in anabolic efficacy, in which protein composition can be used to calculate various protein scores such as the digestible indispensable amino acid score (DIAAS). As collagen protein and collagen protein hydrolysates (CP) lack tryptophan, the assigned DIAAS score is 0 and therefore CP benefit by being supplemented with tryptophan or blended with complete proteins such as MP. However, despite CP being assigned of poor protein quality, there are reports of CP having biological effects. In some studies, CP have been shown to reduce nitrogen excretion and maintain body weight in supplemental trials of energy restriction and exercise [21], while having no effects in others [22]. Such effects of collagen-derived peptides may seem counter-intuitive in light of them being primarily non-essential amino acids (NEAA) by sequence, despite proposed anabolic signaling properties in cell culture [23]. Given our previous work illustrating the limited leucine requirement to robustly stimulate MPS in older adults, at rest and after resistance exercise (RE; [16]), coupled to the proposed anabolic properties of collagen peptides/NEAA [23,24]; we investigated the effects of a low volume protein blend of CP and milk proteins (CP+MP), compared to a 100% milk-protein blend (MP), in older individuals at rest and after RE. It was hypothesized that the CP blend would provide an equivalent stimulation of MPS, due principally to its leucine content, both at rest and in response to RE.

## 2 Materials and methods

### 2.1 Subject characteristics and ethics

This study was performed according to the Declaration of Helsinki. Following ethical approval, granted by the University of Nottingham Medical School Ethics Committee, eight older men were recruited from the local geographical area via postal advertisement (see Table 1 or subject demographics). Sample size was powered based on the primary end point of acute MPS. Based on previous data from our lab investigating feeding responses to a range of protein intakes [25], using a pooled SD of acute MPS measures of 0.022, a sample size of 8 in each group would be able to detect a difference in fasted to fed MPS >30% at a >80% power (based on a Cohen's d estimation of effect size of 1.4 using a two-group unpaired t-test with a 0.05 two-sided significance. Men only were studied here simply since the small but adequate single-sex sample size (upon which the study was powered), may be confound by mixed-sex responses. To minimize confounding variables, exclusion criteria included: a BMI <18 or >32 kg·m^−2^, active cardiovascular disease: uncontrolled hypertension (BP > 160/100), angina, heart failure (class III/IV), arrhythmia, right to left cardiac shunt or recent cardiac event, cerebrovascular disease: previous stroke, aneurysm (large vessel or intracranial), respiratory disease including pulmonary hypertension or COPD, metabolic disease: hyper- or hypoparathyroidism, untreated hyper- or hypothyroidism, Cushing's disease, type 1 or 2 diabetes (treated and untreated), inborn/congenital errors of metabolism (e.g. PKU, galactosaemia), active inflammatory bowel disease, acute or chronic renal disease, malignancy (or history of malignancy with 5 y), recent steroid treatment (within 6-months) or hormone replacement therapy, coagulopathy, musculoskeletal or neurological disorders. Following recruitment and provision of written informed consent, and before inclusion in the project, all volunteers were screened by a physician (at least one week prior to the study day) by means of a medical questionnaire, physical examination and resting ECG, to exclude for any condition outlined above or other symptoms of ill health. All volunteers had normal blood chemistry, were normotensive (BP < 140/90), performed activities of daily living and recreation but were not involved in any formal exercise regimes, and were not on a weight-controlling diet. Muscle mass and strength of the lower limbs plays a crucial role in functionality and the development of frailty. As such, many studies focus on the effects of RE and nutritive interventions on leg muscle adaptability. During screening knee extensor strength was assessed via a one repetition maximum assessment (1-RM), using the volunteer's dominant leg (Technogym, Gambettola, Italy).

**Table 1:** Subject demographics n = 8.

Age (y)	Height (cm)	Weight (kg)	BMI (kg/m^2^)	1-RM (kg)	Leg LM (kg)	Total LM (kg)	BF (%)
71 ± 1	174 ± 2	81 ± 3	27 ± 1	41 ± 1	17 ± 2	53 ± 2	32 ± 4

Data is presented as mean ± standard error of the mean SEM, BMI: body mass index; LM: lean mass; BF: body fat.

### 2.2 Study procedure

**Fig. 1:**
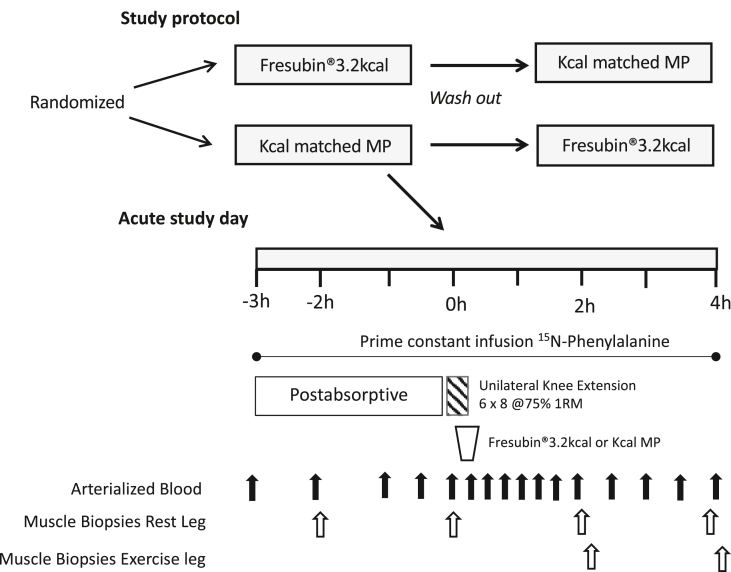
Study Protocol. Study protocol: effects of Fresubin®3.2 kcal DRINK or a kcal matched MP at rest and after resistance exercise in older men. 1RM, one repetition maximum, MP, milk protein.

This study was a blinded crossover trial with subjects reporting to the laboratory on two separate occasions (≧ 7 day washout period) to undertake the same study procedures in which they were randomly assigned to receive either 1 bottle of Fresubin®3.2 kcal (CP+MP) (400 kcal, 20 g Protein, 35 g Carbohydrate, 20 g Fat, 125 ml) or 1 bottle of a kcal matched MP (400 kcal, 20 g Protein, 45 g Carbohydrate, 15.6 g Fat, 200 ml) (Fig. 1). Study drinks were matched for protein and energy intake with drink volumes reflecting the difference in energy density of Fresubin®3.2 kcal compared to other ONS. On the first study visit only, body composition was assessed via Dual X-ray Absorptiometry (DXA). For each of the two study visits, subjects arrived in the morning (0800 h) following an overnight fast from 2000h the previous day (water *ad libitum*). To quantify MPS, volunteers had a cannula inserted into the antecubital vein of one arm for the infusion of a ^15^N-Phenylalanine tracer (Isotec, Sigma Aldrich; prime: 0.4 mg·kg^−1^, constant: 0.6 mg·kg^−1^ h^−1^), with a retrograde cannula inserted into the dorsal capillary bed of the hand to sample arterialized blood. Muscle biopsies were taken −2 h and 0 h after commencement of tracer infusion to permit the assessment of basal (post-absorptive) MPS. Volunteers then performed a bout of unilateral knee-extension exercise previously shown [26] to maximally stimulate MPS (6 × 8 repetitions at 75% of their pre-determined 1-RM using the volunteer's dominant leg with 2 min inter-set rest period). If the volunteer failed to complete 8-repetitions, then the inter-set break was allowed before moving on to the next set. Immediately following the exercise, each volunteer consumed either CP+MP or a kcal matched MP. This unilateral study design meant that the non-exercised leg was exposed to the effect of the feed (‘FED’), while the exercised leg was exposed to the combination of feeding and exercise (‘FED-EX’). Further muscle biopsies were then taken 2 h and 4 h after feeding to permit the assessment of muscle anabolic signalling, with MPS over the 0–4 h period. Muscle biopsies were collected from the vastus lateralis (VL) using the conchotome technique [27] after induction of local anaesthesia via infiltration of 5 ml 1% lignocaine. Biopsies were washed with ice-cold phosphate buffered saline before being snap frozen in liquid N_2_ and stored at −80 °C until analysis.

### 2.3 Measurement of plasma insulin and AA concentrations

Plasma insulin concentration was measured using a high-sensitivity human insulin enzyme-linked immunosorbent assay (ELISA) in accordance with manufacturers instruction (DRG Instruments GmbH, Marburg, Germany). For plasma AA analyses, 10 μl of a mix of stable isotopically labelled internal standards were added to 200 μl of plasma, treated with urease and deproteinised with 1 ml ice cold ethanol on ice for 20 min. Following centrifugation (17,000×*g*; 5 min), the supernatant was decanted and evaporated to dryness under nitrogen. Following resuspension in 0.5 M HCl, lipids were extracted with 2 ml of ethyl acetate before the lower HCl phase was evaporated and AA were derivatized to their tBDMS esters. AA concentrations were quantified against a standard curve of known concentrations using GC–MS (Agilent).

### 2.4 Measurement of MPS

To determine bound ^15^N-Phenylalanine, myofibrillar proteins were isolated, hydrolysed and derivatized using our standard techniques [4]. Briefly, ~25 mg of muscle biopsy tissue was homogenized in ice-cold homogenization buffer (50 mM Tris–HCl (pH 7.4), 50 mM NaF, 10 mM B-glycerophosphate disodium salt, 1 mM EDTA, 1 mM EGTA, 1 mM activated Na3VO4 (all Sigma Aldrich, Poole, UK)) and a complete protease inhibitor cocktail tablet (Roche, West Sussex, UK) at 10 ml/mg of tissue. Following centrifugation at 13,000 g for 5 min at 4 °C, the resulting insoluble pellet was washed three times with homogenization buffer to remove excess free AA and solubilized in 0.3 M NaOH to aid separation of the soluble myofibrillar fraction from the insoluble collagen fraction by subsequent centrifugation. The soluble myofibrillar fraction was precipitated using 1 M perchloric acid (PCA), pelleted by centrifugation and washed twice with 70% ethanol. The protein-bound AA were released by acid hydrolysis using 0.1 M HCl and 1 ml of Dowex ion-exchange resin (50 W-X8-200) overnight at 110 °C. The free AA were purified, derivatized and the fractional synthesis rate (FSR) of the myofibrillar proteins was calculated using the precursor–product equation below: FSR = [ΔEm/(Ep × t)] × 100, where ΔEm is the change in enrichment of bound ^15^N phenylalanine in two sequential biopsies, t is the time interval between two biopsies in hours, and Ep is the mean free ^15^N phenylalanine enrichment in the intramuscular pool. Muscle intracellular phenylalanine enrichment was measured by gas chromatography-mass spectrometry (Trace DSQ; Thermofisher Scientific) following precipitation of the sarcoplasmic fraction and purification of the aqueous supernatant using Dowex H^+^ resin, with AAs converted to their *tert*-butyldimethylsilyl derivatives.

### 2.5 Immunoblotting for anabolic signalling pathway activity [phosphorylation]

Immunoblotting was performed as previously described [28] using the sarcoplasmic fraction isolated from MPS preparation. Sarcoplasmic protein concentrations were analysed using a NanoDrop ND1000 spectrophotometer (NanoDrop Technologies, Inc., Wilmington, DE-US) and sample concentrations adjusted to 1 μg/μl in 3x Laemmli buffer to ensure equivalent protein loading onto pre-cast 12% Bis-Tris Criterion XT gels (BioRad, Hemel Hempstead, UK) of 10 μg/lane. Samples were separated electrophoretically at 200 V for 1 h, followed by wet transfer of proteins to PVDF membrane at 100 V for 45 min and subsequent blocking in 2.5% non-fat milk in 1 Tris buffered saline/Tween 20 (TBST) for 1 h. Membranes were incubated in primary antibodies (1:2000 dilution in 2.5% BSA in TBST) overnight at 4 °C; p-mTOR Ser 2448 (#2972), p-EEF2 Thr56 (#2331), p-AKT Ser473 (#4060) (New England Biolabs, Hertfordshire, UK). Membranes were subsequently washed and incubated in HRP conjugated anti-rabbit secondary antibody (#7074, New England Biolabs, Hertfordshire, UK; 1:2000 in 2.5% BSA in TBST) at ambient temperature for 1 h, before being exposed to chemiluminescent HRP Substrate (Millipore Corporation, Billerica, MA-US) for 5 min and bands quantified by Chemidoc XRS (BioRad, Hertfordshire, UK). All signals were within the linear range of detection; loading was corrected to Coomassie [34].

### 2.6 Statistical analyses

Data are presented as means ± standard error of the mean (SEM). Data were checked for normal distribution using a Kolmogorov Smirnov Test. Plasma AA, insulin, MPS and immunoblotting were analysed using a repeated measures two-way ANOVA. The insulin AUC and percent change in MPS was analysed using a paired t-test. All data analysis was performed using GraphPad Prism (GraphPad Software Inc, San Diego, CA); the alpha level of significance was set at P < 0.05.

## 3 Results

### 3.1 Plasma AA and insulin concentrations

**Fig. 2:**
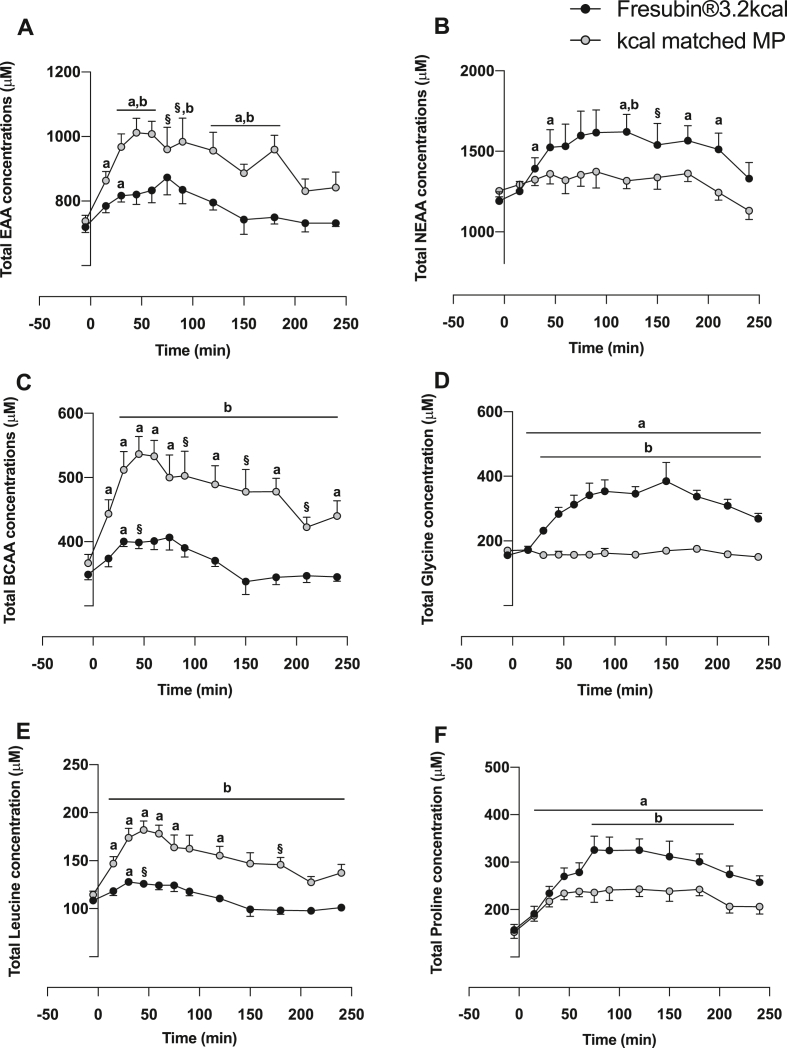
Plasma AA concentration. Time course effects of Fresubin®3.2 kcal DRINK or kcal matched MP on plasma concentrations of A) total EAA, B) total NEAA, C) total BCAA, D) total glycine, E) total Leucine and F) total proline. ^a^ Significantly different from baseline (P < 0.05). ^§^ Trend for greater than baseline 0.05< P < 0.1 ^b^ Significantly different than other group at the specific time point indicated (P < 0.05).

**Fig. 3:**
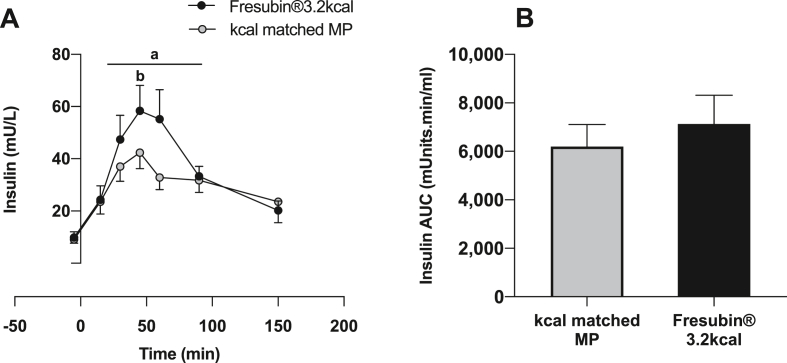
Plasma Insulin. Time course effects of Fresubin®3.2 kcal DRINK or kcal matched MP on A) plasma insulin concentrations and B) plasma insulin AUC. ^a^ Significantly different from baseline (P < 0.05). ^b^ Significantly different than other group at the specific time point indicated (P < 0.01).

Plasma EAA (Fig. 2A), branch chain amino acids (BCAA) (Fig. 2C) and leucine (Fig. 2E) concentrations increased rapidly in response to MP, peaking at 30–60 min (all P < 0.01). Similarly, plasma EAA, BCAA and leucine also increased in response to CP+MP peaking at ~30 min (P < 0.01). As expected, the MP drink showed significantly greater increases in plasma EAA (MP mean: 917 ± 25 μM, CP+MP mean: 786 ± 15 μM (Feed P < 0.01), BCAA (MP mean: 475 ± 25 μM, CP+MP mean: 372 ± 15 μM (Feed P < 0.001) and leucine (MP mean: 152 ± 6 μM, CP+MP mean: 113 ± 4 μM (Feed P < 0.001) concentrations which remained elevated above baseline for 150–200 min. Plasma NEAA only increased in response to CP+MP (Fig. 2B), with glycine (Fig. 2D) and proline (Fig. 2F) increasing rapidly in responses to CP+MP, peaking at ~75–100 min and remaining elevated for ~250min (all P < 0.05). Plasma insulin concentration increased over postabsorptive values in response to both drinks, peaking at 42 ± 6 mU/mL with MP (P < 0.01) and 58 ± 10 mU/mL with CP+MP (P < 0.01); as such being significantly greater with CP+MP (P < 0.01) (Fig. 3A). Despite this, there was no difference in the area under the curve (AUC) for insulin in response to MP (AUC: 6194 ± 906) vs. CP+MP (AUC: 7172 ± 1186) (Fig. 3B).

### 3.2 Skeletal muscle protein synthesis (MPS)

**Fig. 4:**
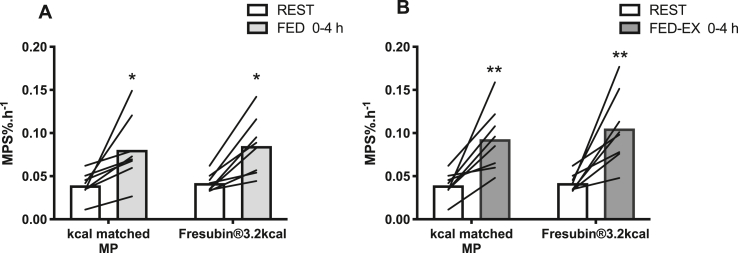
Muscle protein synthesis. The effect of Fresubin®3.2 kcal DRINK or kcal matched MP on muscle protein synthesis (MPS) in A) FED 0–4 h and B) FED-EX 0–4 h conditions. ∗ Significantly different from rest (P < 0.05), ∗∗ (P < 0.01).

In the FED leg, MPS determined via direct incorporation into muscle biopsy techniques increased similarly in response to MP (0.039 ± 0.005%/h to 0.081 ± 0.014%/h, P < 0.05) and CP+MP (0.042 ± 0.004%/h to 0.085 ± 0.007%/h, P < 0.05) over 0–4 h (Fig. 4A), with no difference in MPS increases between the drinks (MP: 116 ± 38% vs. CP+MP: 100 ± 20%). Similarly, in the FED-EX leg, MPS increased over 0–4 h with MP (0.039 ± 0.005%/h to 0.093 ± 0.013%/h, P < 0.01) (Fig. 4B) and with CP+MP (0.042 ± 0.004%/h to 0.105 ± 0.015%/h, P < 0.01), with no difference in MPS increases between the drinks (MP: 166 ± 44% vs. CP+MP: 157 ± 37%).

### 3.3 Muscle anabolic signalling

**Fig. 5:**
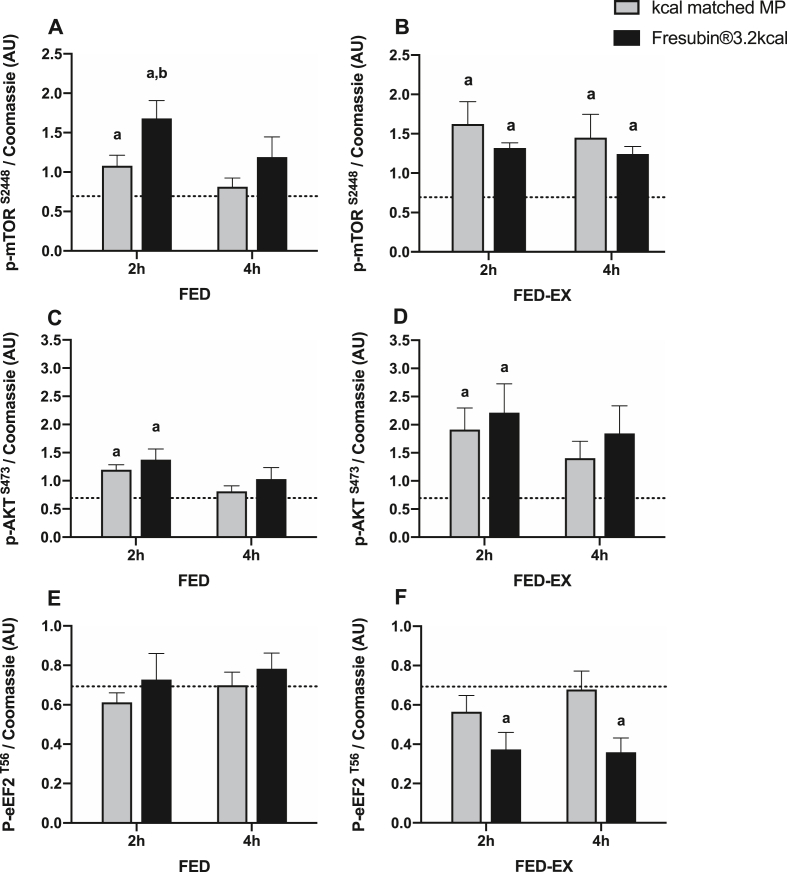
Anabolic Signaling. The effect of Fresubin®3.2 kcal DRINK or kcal matched MP on muscle signalling responses to A) p-mTOR FED, B) p-mTOR FED-EX, C) p-AKT FED, D) p-AKT FED-EX, E) p-eEF2 FED and F) p-eEF2 FED-EX. Responses were normalised to baseline and log transformed. Dotted line represents baseline. ^a^ Significantly different from baseline (P < 0.05). ^b^ Significantly different than other group at the specific time point indicated (P < 0.05).

To assess anabolic signalling relating to the control of MPS we measured the phosphorylation of key anabolic substrates including the mechanistic target of rapamycin complex 1 (mTORC1), protein kinase B (AKT) and the eukaryotic elongation factor 2 (eEF2). In the FED leg, both MP and CP+MP significantly elevated mTOR activation at 2 h (P < 0.05), with the response to CP+MP being significantly greater (P < 0.05) (Fig. 5A). Protein kinase B (AKT) activation was also elevated at 2 h in response to both MP and CP+MP, whilst there was no change in eEF2 activation at any time-point (Fig 5C and E). In the FED-EX leg, both MP and CP+MP significantly elevated mTOR activation at 2 and 4 h (P < 0.05) (Fig. 5B). AKT activation was elevated at 2 h in the FED-EX leg in response to both MP and CP+MP (P < 0.05) (Fig. 5D). There was a significant decrease in eEF2 phosphorylation, representing increased activation, at 2 and 4hrs with CP+MP only (P < 0.05) (Fig. 5F).

## 4 Discussion

The gradual loss of muscle mass in ageing (and ill-health) is associated with the development of anabolic resistance, representing a diminished MPS response to the anabolic stimuli of nutrition and exercise [29,30]. As such, there is increasing evidence that older individuals require greater levels of protein intake to enhance MPS responses [8]. Increasing protein intake in fragile cohorts can be of great difficulty due to decreased appetite and increased satiety, with oral nutritional supplement (ONS) volume strongly affecting compliance [31]. CP have shown longer-term anabolic efficacy in both young and older cohorts [24,32] and stimulation of anabolic signalling activities [23]. Further, CP based low volume, high energy/protein ONS have shown promise in acceptability and compliance [20], characteristics that may be advantageous for ONS. Nonetheless, accepting the presumed negligible role of NEAA in regulating acute MPS, we sought to investigate the anabolic properties of Fresubin®3.2 kcal DRINK (a CP+MP blend) versus a 100% MP supplement.

It is now well established that the EAA components of a protein meal drive increases in MPS, with optimal protein/EAA quantity, quality and delivery having undergone substantial research [14,18,33]. A clear and consistent outcome of these studies is that only modest amounts of protein, rich in EAA, or free EAA, are required to robustly stimulate MPS [16,25]. In fact, we (and others) have shown that of all the AA, leucine can robustly stimulate MPS [4] and in part these effects are exerted by directly targeting mTOR signaling pathways [28,34]. It has been suggested that a threshold of 3 g of leucine is required to maximally stimulate MPS; however we have shown that EAA mixtures containing < 3 g of leucine are sufficient to maximally stimulate MPS [16,25] - at least in older women. Further, in young males, a suboptimal dose of whey protein containing either 0.75 g or 3 g of leucine produced equivalent increases in FED MPS [35]. However, with the anabolic potency of leucine well recognised, proteins low in EAA/leucine such as collagen are typically considered to be of lesser biological value [13]. In this study, despite the greater CP content in the CP+MP blend and reduced EAA appearance than the MP drink, CP+MP resulted in significant increases in plasma EAA/leucine-albeit to a lesser extent than that of MP. The majority of EAA/leucine would be expected to originate from the MP content; however, in being a protein blend, the exact contributions of CP and MP cannot be defined. For instance, consumption of hydrolyzed collagen alone can significantly increase EAA and leucine 20–60 min after consumption [36]. However when compared to an equivalent bolus of whey protein, plasma EAA and leucine resulting from CP alone are discernably different [37].

The precise amino acid quantity and composition required to stimulate MPS is not definitive. For instance, we have shown providing 3 g of the single amino acid leucine can increase MPS without provision of other AA [4]. As such, despite CP being an incomplete protein, increased plasma leucine after CP consumption [36] may theoretically impact MPS – depending upon the level of intake. Comparisons of CP to animal proteins in various settings have been trialed. A CP supplement containing 0.9 g of leucine, in a unilateral RE design, illustrated increases in acute MPS with RE + CP supplementation, yet this was significantly less vs. whey protein and not sustained chronically [37]. Similarly, CP supplementation combined with high intensity interval training (HIIT) increased 4 day MPS - albeit significantly less so than whey (lactalbumin) [38]. In the present study, we found that the blend of CP+MP produced equivalent increases in FED-state MPS as the kcal matched MP. The observed MPS response, likely driven by increases in EAA/leucineamia, would ostensibly be driven by the presence of MP. This is reflected by post-absorptive AA profiles being similar to those previously reported after consumption of small amounts of EAA resulting in robustly stimulated MPS in older women, despite lower EAA loads [16]. Agreeing with previous work, peak activation of MPS may not be exclusively driven by leucinaemia; for instance, AA other than leucine have been shown to stimulate MPS [2].

Interestingly, the CP+MP blend produced a greater increase in mTOR signaling despite containing lower leucine content. This may be a result of collagen-peptides, with the novel food derived collagen-peptides prolyl-hydroxyproline (Pro-Hyp) and hydroxyprolyl-glycine (Hyp-Gly) having been identified in plasma after CP consumption [39]. In particular, Hyp-Gly has shown anabolic potency on C2C12 muscle cells, in vitro, increasing myotube diameter and activating AKT/mTOR signalling [23]. Our primary aim was not to investigate the specific effects of Pro-Hyp and Hyp-Gly on MPS and therefore we did not measure plasma appearance. However, the effect of individual collagen peptides on human muscle anabolism remains unclear despite promising in vitro data and therefore warrants further study. In addition to containing bioactive peptides, CP supplementation contains significant amounts of NEAA such as glycine and proline that were substantially increased in the bloodstream after CP+MP consumption. Glycine in particular has shown efficacy at stimulating MPS and AKT/mTOR signalling in C2C12 muscle cells and therefore higher NEAA may potentiate anabolic signalling [40]. However, CP+MP resulted in a greater peak insulin concentrations that is known to act as a powerful stimulator of mTOR phosphorylation [41]. As such, the greater relative increase in mTOR phosphorylation could reflect the unique composition of CP + MP or the fact that some NEAA such as glycine act as mild insulin secretagogues [42].

Resistance exercise training (RET) represents one of the foremost countermeasures against age-related loss of muscle mass [43], despite the anabolic responses to both nutrition and exercise becoming blunted in older age [6,26,44]. As such, it is of importance to optimise nutritional approaches to maximise anabolic responses to RET. Despite being an incomplete protein source, CP supplements in combination with RET have shown efficacy at increasing fat-free mass in pre-menopausal women [45], sarcopenic men [24] and young recreationally active men [46] (vs. placebo). Further, CP supplementation was shown to be more effective than whey at reducing nitrogen excretion and preserving body mass during a low protein diet [21]. As such, there is evidence that CP-derived proteins may have a supportive role in addition to those of EAA load, in human muscle. The present study demonstrated FED-EX MPS responses as equal between CP+MP and MP, demonstrating a low level of EAA combined with a full complement of AA can robustly stimulate MPS. The activation of AKT/mTOR was also equivalent; however interestingly, eEF2 activity (indicated by decreased phosphorylation promoting mRNA elongation phases of translation) was increased with RE only in the CP+MP group. Heightened eEF2 activation with RE and CP+MP could be a result of elevated insulin [47] or effects of heightened NEAA on anabolic signalling [40]. Proteomic analysis of CP in combination with RET has shown an increase in the level of proteins related to mRNA translation [32]. As such, CP sources of AA/peptides may have effects on anabolic signalling that warrant further investigation. In both FED, and FED-EX scenarios, it is plausible that the MP content of the CP+MP blend drink is sufficient to bring about the equivalent MPS responses reported here. However, altered anabolic signaling is seen in FED (mTOR) and FED-EX (eEF2) conditions, indicating possible bio-active roles of CP. The temporal relationship between indicators of activated MPS and direct measures of MPS has shown a level of dissociation [48,49] and therefore extrapolation of these findings can be limited. However, previous reports of specific collagen-peptides such as Pro-Hyp/Hyp-Gly and/or elevated NEAA such as glycine enhancing anabolic signalling (and which also have been shown to have protective effects during muscle wasting [50]) and or insulin that, with time, may have additional benefits on skeletal muscle health.

Finally, achieving sufficient calorie and protein intake is a great challenge in malnutrition, and a key factor in this is the volume of the ONS - which greatly affects compliance [19]. Crucially, the volume of CP+MP was significantly reduced (i.e. 125 ml vs 200 ml in MP) in comparison to MP, whilst providing equivalent MPS responses. Further, CP+MP demonstrated augmented anabolic signalling that may have long term anabolic benefits. For instance, nitrogen excretion was reduced and body mass maintained when consuming CP vs whey protein during a low protein diet [21]. Nutritional composition and the balance between total nitrogen and EAA content may therefore play a significant role in fighting malnutrition and inactivity-significant clinical problems associated with age and disease related anabolic resistance. That being said, we did not study these effects in young adults and therefore further studies are required to determine any acute or long-term benefits of CP in ageing and disease. In addition, there are many aspects to protein consumption that may affect anabolic efficiency, including digestibility, bioavailability and tissue utilization. Advances in stable isotope techniques, such as the dual isotope method to asses digestibility [51] are beginning to inform on the true digestibility, bioavailability and tissue utilization of proteins, thereby providing a more holistic view of nutritional biology. To conclude, Fresubin®3.2 kcal DRINK, through EAA/leucine content, and/or aspects of its unique MP/CP blended composition, may provide a suitable protein/energy dense ONS to mitigate muscle mass loss in older adults.

## Statement of authorship

PJA, KS, BEP, conceived and designed the study. PS, MSB, SW performed data collection. MSB, SW, DJW, BEP, PS, JC & JB performed the sample processing, data analyses and construction of figures. All authors contributed to the preparation and drafting of the final manuscript.

## Funding sources

The current work was funded via an investigator initiated Fresenius-Kabi grant to BEP and PJA and was also supported by the 10.13039/501100000265Medical Research Council (grant numbers MR/R502364/1 and MR/P021220/1) as part of the MRC-Versus Arthritis Centre for Musculoskeletal Ageing Research awarded to the Universities of Nottingham and Birmingham, and the National Institute for Health Research, Nottingham Biomedical Research Centre.

## Conflict of interest

PJA has acted as a consultant to Fresenius-Kabi. All other authors declare no potential conflict of interest.
